# Cardio-pathogenic variants in unexplained intrauterine fetal death: a retrospective pilot study

**DOI:** 10.1038/s41598-021-85893-0

**Published:** 2021-03-24

**Authors:** Dana A. Muin, Martina Kollmann, Jasmin Blatterer, Gregor Hoermann, Peter W. Husslein, Ingrid Lafer, Erwin Petek, Thomas Schwarzbraun

**Affiliations:** 1 Division of Fetomaternal Medicine, Department of Obstetrics and Gynecology, Medical University of Vienna, Waehringer Guertel 18-20, 1090 Vienna, Austria; 2 Division of Reproductive Medicine, Department of Obstetrics and Gynecology, Medical University of Graz, Graz, Austria; 3 Institute of Human Genetics, Medical University of Graz, Graz, Austria; 4 Department of Laboratory Medicine, Medical University of Vienna, Vienna, Austria; 5 MLL Munich Leukemia Laboratory, Munich, Germany; 6 Department of Internal Medicine, General Hospital Muerzzuschlag, Mürzzuschlag, Austria

**Keywords:** Genetics research, Genetics

## Abstract

To describe the prevalence and spectrum of cardio-pathogenic variants in singleton fetuses after unexplained intrauterine fetal death (IUFD). DNA from post-mortem fibroblastic tissue samples of 16 fetuses after unexplained IUFD was retrieved at two tertiary university hospitals for clinical exome sequencing with subsequent filtering of 122 cardio-specific genes to elucidate underlying cardio-pathogenic variants. In total, we included 12 (75%) male and four (25%) female fetuses who were stillborn at a median gestational age of 34^+6^ (23^+2^–40^+5^) weeks. In two (12.5%) fetuses no cardio-pathogenic variants were found. In 14 (87.5%) fetuses, overall 33 variants were detected in 22 cardio-specific genes, involving 14 (63.63%) genes associated with cardiomyopathy, six (27.27%) arrhythmogenic susceptibility genes and two (9.09%) arrhythmia and cardiomyopathy associated genes. Among the 33 variants, five (15.2%) were classified as *likely benign* according to the *American College of Medical Genetics and Genomics*; 28 (84.8%) variants were considered as *variants of uncertain significance*. Compared to a cohort of explained IUFDs, the cases with and without fetal variants in cardiac genes differed not significantly regarding maternal age, previous history of stillbirth, time of stillbirth or fetal sex. Unexplained stillbirth may be caused by cardio-genetic pathologies, yet a high number of variants of *uncertain significance* merit a more detailed post-mortem examination including family segregation analysis.

## Introduction

Intrauterine fetal death (IUFD) is a devastating event which warrants a thorough investigation in order to elucidate the underlying pathophysiology. It is essential that parents receive comprehensive and accurate facts about risk factors and causes of the death of their child, as it supports them in their grieving process and provides valuable information regarding possible recurrence risk in future pregnancies. Gold standards in post-mortem investigations include conventional fetal autopsy, placental histology and maternal examinations. Genetic analyses, such as chromosomal microarray analysis, have been proved valuable in providing a cause for the perinatal demise and are usually incorporated into the fetal post-mortem work-up^1–3^. Fetal deaths are termed as “unexplained” in cases where no deleterious factor has been found despite thorough post-mortem examinations^4^. The incidence of unexplained IUFD has been estimated between 14 and 47%^5,6^.

Previous studies have suggested the influence of cardiac arrhythmias as a possible cause for fetal death in phenotypically normal fetuses and without detectable myocardial or brain lesions in autopsy^7,8^. To date, long-QT syndrome (LQTS) is one of the most investigated genetic cardiac disorders that has been associated with arrhythmias as early as in the fetal period and has been previously found in up to 10% of otherwise unexplained stillbirths^9,10^.

By this study, we aimed to elucidate the prevalence and spectrum of cardio-pathogenic variants in fetuses whose cause of death had remained unknown despite thorough post-mortem investigation, including placental histology, fetal autopsy, microarray and chromosomal analysis, as well as maternal tests. We assumed a prevalence of up to 50% for an underlying genetic disease that may have contributed to the fetal demise. To prove our hypothesis we retrieved archived DNA samples of a selected cohort of phenotypically normal fetuses whose cause of death remained unexplained. We subsequently analyzed 122 genes responsible for cardiac function and/or morphology by targeted sequencing in these subjects.

## Methods

### Study design and data collection

**Figure 1:**
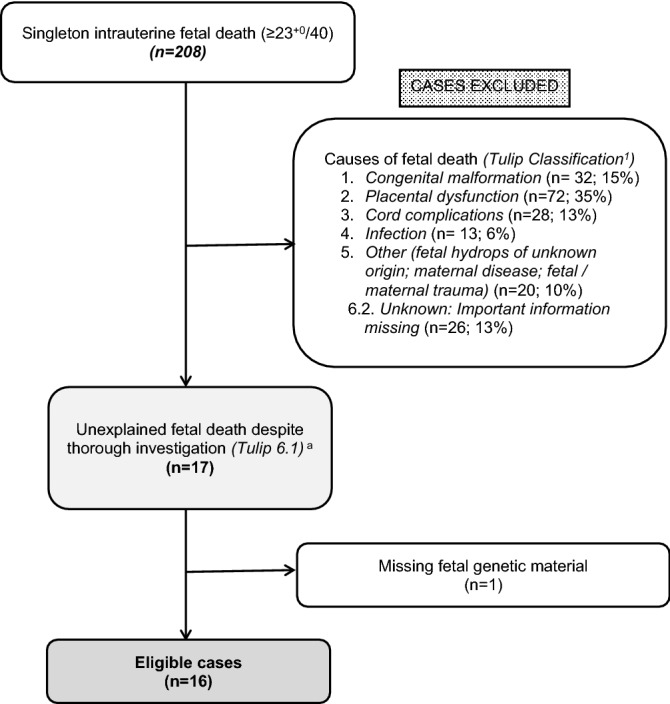
Flowchart showing selection process of cases between January 2003 and December 2017. Superscript a: Investigation includes fetal post-mortem examinations (fetal autopsy; post-mortem magnetic resonance imaging; karyotyping and microarray), maternal examinations (laboratory tests) and placental histology. Korteweg et al.^11^.

We retrospectively reviewed all cases of intrauterine fetal death delivered at the Medical University of Vienna, Austria, and the Medical University of Graz, Austria, between January 2003 and December 2017. Cause of fetal death had been defined as the “*initial, demonstrable pathophysiological entity initiating the chain of events that has irreversibly led to death*” and had been categorized according to the Tulip classification upon review of guideline-based post-mortem examinations including fetal autopsy, postmortem Magnetic Resonance Imaging, fetal genetics (karyotyping and microarray), placental histology, maternal examinations and clinical perinatal data^11^. For routine genetic analysis, post-mortem fetal gluteal muscle tissue had been obtained under sterile conditions straight after stillbirth and stored in 10 ml of sterile 0.9% saline solution. Only cases classified as “unknown cause of fetal death despite thorough investigation” with archived fetal DNA were further analyzed for this study (Fig. 1).

### Definitions

Ethnicity was self-reported by the pregnant woman at time of antenatal booking. Maternal age was defined as age in years at the time of delivery, parity was defined as the number of previous births at time of antenatal booking. Body mass index (BMI) at booking was grouped as underweight (< 18.9 kg/m^2^), normal (19.0–24.9 kg/m^2^), overweight (25.0–29.0 kg/m^2^) and obese (≥ 30.0 kg/m^2^). Smoking was defined as current smoker or non-smoker at the time of antenatal booking. Consanguinity and women’s medical and family history were retrieved from the electronic medical records using ViewPoint Version 5.6.28.56 (General Electric Company, Solingen, Germany). Fetal data collection involved fetal sex, weight in grams (g) and length in centimeters (cm). Autopsy reports and placental histology were derived from the hospital data system SAP GUI for Windows Version 7500.2.6.3379 (SAP NetWeaver, Austria) or collected manually from the archived medical records.

### DNA extraction and quality validation

Fetal DNA from biopsied muscle tissue was isolated using standard techniques. For quality assessment after DNA-isolation we used photometric methods (Agilent Bioanalyser and NanoDrop 3300 fluorometer; ThermoFisher Scientific). Only samples with sufficient quality were processed further using the Next-generation sequencing enrichment panel.

### Target enrichment and sequencing

For the selected samples with sufficient DNA quality we used the TruSight One Sequencing Panel (Illumina) covering 4813 genes with known disease associations. Enrichment was performed according to the supplier’s instructions and the resulting libraries were sequenced on a NextSeq Sequencer (Illumina) according to standard protocols.

### Data analyses

Data were analyzed with a well-established bioinformatics pipeline for routine diagnostics: First, the obtained sequencing results were filtered for 122 genes that are known to be associated with specific cardiac phenotypes (Supplementary Table 1). For our target genes, only protein-coding exons and flanking intronic sequences with coverage of at least 20 were analyzed further. We did not evaluate any variants with potential implications on other phenotypes, which were not subject of this study. After trimming, alignment and quality assessment, existing variance from the reference genome was analyzed using the VariantStudio Software by Illumina and annotated to existing databases. Annotated variants were filtered according to their allelic frequency in the Genome Aggregation Database (Version 2) and those occurring in less than 1% were analyzed further. All rare variants were checked for plausibility using the Integrative Genomics Viewer Software (Broad Institute and the Regents of the University of California) to evaluate whether the variant was truly present and correctly annotated. We then checked the variants’ relevance and investigated whether the observed frequency was consistent with the suspected phenotype and whether entries already existed in mutation databases and had been described in literature before^12^. Variant assessment occurred at two time points: initial classification after exome analysis (December 2018) and during reclassification within this manuscript’s revision (November 2020). Where no information on a specific variant was available, we evaluated its effect on the gene product and its potential impact on gene function and potential lethality.

**Table 1:** Summary of the 33 variants detected in 22 genes in 14 cases of otherwise unexplained intrauterine fetal demise.

Gene	Gene description	Primary dysfunction	Location	Nucleotide change	Amino acid change	Effect	RefSeq	ACMG classification	ACMG criteria	VarSome in silico prediction
*AKAP9*	A-Kinase Anchoring Protein 9	Arrhythmogenic	7q21-q22	c.763A > C	p.Thr255Pro	Missense	NM_005751.4	VUS	PM2	12 benign, 8 damaging
*ANK2*	Ankyrin 2	Arrhythmogenic	4q25-q27	c.7436A > G	p.Lys2479Arg	Missense	NM_001148.4	VUS		12 benign, 7 damaging
				c.11459G > A	p.Arg3820Gln	Missense	NM_001148.4	Likely benign	BS1, BP6, BP4	17 benign, 3 damaging
				c.9520A > G	p.Thr3174Ala	missense	NM_001148.4	VUS	PM2, BP4	19 benign, 1 damaging
*DSG2*	Desmoglein 2	Cardiac morphology, arryhthmogenic	18q12.1	c.3205A > G	p.Met1069Val	missense	NM_001943.3	VUS	PM2, BP4	20 benign, 0 damaging
				c.880A > G	p.Lys294Glu	missense	NM_001943.3	VUS		11 damaging, 9 benign
*DSP*	Desmoplakin	Cardiac morphology	6p24	c.5513G > A	p.Arg1838His	missense	NM_004415.2	VUS	BP1	10 damaging, 10 benign
*ILK*	Integrin-linked kinase	Cardiac morphology	11p15.5-p15.4	c.521G > A	p.Arg174His	missense	NM_004517.2	VUS		8 benign, 7 damaging
				c.684 T > G	p.Ser228Arg	missense	NM_004517.2	VUS	PM2	12 benign, 8 damaging
*JPH2*	Junctophilin 2	Cardiac morphology	20q13.12	c.572C > G	p.Pro191Arg	missense	NM_020433.4	Likely benign	BS1, BP4	18 benign, 2 damaging
*KCNE1*	Potassium voltage-gated channel subfamily E member 1	Arrhythmogenic	21q22.12	c.200G > A	p.Arg67His	missense	NM_001270402.1 NM_000219.6 (HGMD; ClinVar)	VUS	PM2, PM5, PP3	12 damaging, 3 benign
*LAMA2*	Laminin alpha 2 (merosin)	Cardiac morphology	6q22-q23	c.6598C > T	p.Arg2200Cys	missense	NM_000426.3	VUS	PP3, BP1	17 damaging, 2 benign
				c.3778G > A	p.Glu1260Lys	missense	NM_000426.3	VUS	PM2, BP1	12 damaging, 7 benign
*MYH7*	Myosin heavy polypeptide 7	Cardiac morphology	14q12	c.3200 T > C	p.Met1067Thr	missense	NM_000257.2	VUS	PM2, PP2, PP3	17 damaging, 3 benign
				c.2224G > A	p.Ala742Thr	missense	NM_000257.2	VUS/Likely pathogenic	PM1, PM2, PP2, BP4	14 benign, 5 damaging
*MYO6*	Myosin VI	Cardiac morphology	6q13	c.2307C > G	p.Ile769Met	missense	NM_004999.3	VUS	PM2, BP4	15 benign, 6 damaging
*MYOM1*	Myomesin 1	Cardiac morphology	18p11.31	c.4814C > T	p.Ser1605Leu	missense	NM_003803.3	VUS		11 benign, 9 damaging
				c.5045dupA	p.Lys1683GlufsTer16	Duplication (frameshift)	NM_003803.3	Likely benign	BS1, BP6	–
*MYPN*	Myopalladin	Cardiac morphology	10q21.3	c.3589G > A	p.Gly1197Ser	missense	NM_001256267.1 NM_032578.3 (HGMD)	VUS	PM2, PP3	18 damaging, 3 benign
*NDUFV2*	NADH dehydrogenase (ubiquinone) flavoprotein 2	Cardiac morphology	18p11.31-p11.2	c.604G > A	p.Glu202Lys	missense	NM_021074.4	VUS	BP4	14 benign, 6 damaging
*NEBL*	Nebulette	Cardiac morphology	10p12	c.267C > G	p.Tyr89Ter	stop	NM_006393.2	VUS/Likely benign	BS1	-
*OBSCN*	Obscurin, cytoskeletal calmodulin and titin-interacting RhoGEF	Cardiac morphology	1q42.13	c.23631A > T	p.Arg7877Ser	missense	NM_001098623.1 (obscntv2 in HGMD) NM_001271223.2 (ClinVar) NM_052843.3 (OBSCN in HGMD)	VUS	PM2, BP4	17 benign, 3 damaging
				c.4393G > A	p.Glu1465Lys	missense	NM_001098623.1	VUS	BS1, BP4	14 benign, 6 damaging
				c.7457 T > C	p.Val2486Ala	missense	NM_001098623.1	VUS		13 benign, 8 damaging
				c.9545C > T	p.Ala3182Val	missense	NM_001098623.1	VUS	BP4	15 benign, 6 damaging
*PRKAG2*	Protein Kinase AMP-Activated Non-Catalytic Subunit Gamma 2	Cardiac morphology	7q36.1	c.1475 T > A	p.Ile492Asn	missense	NM_016203.3	VUS	PP2, PP3	18 damaging, 2 benign
*RBM20*	RNA binding motif protein 20	Cardiac morphology	10q25.2	c.1451C > T	p.Thr484Ile	missense	NM_001134363.1	Likely benign	BS1, BP4, PP2	13 benign, 3 damaging
*SCN10A*	Sodium voltage-gated channel alpha subunit 10	Arrhythmogenic	3p22.2	c.3674 T > C	p.Ile1225Thr	missense	NM_006514.2	Likely benign	PP3, BS1, BS2	18 damaging, 2 benign
*SCNN1A*	Sodium Channel Epithelial 1 Subunit Alpha	Arrhythmogenic	12p13	c.752G > C	p.Arg251Thr	missense	NM_001159576.1 NM_001038.5 (HGMD, ClinVar)	VUS	BP4, PM2	21 benign, 0 damaging
				c.1618G > A	p.Val540Met	Missense	NM_001159576.1	VUS	BP4, PS3	15 benign, 4 damaging
*TBX5*	T-Box Transcription Factor 5	Cardiac morphology, arryhthmogenic	12q24.1	c.1115C > T	p.Ser372Leu	Missense	NM_181486.2 NM_000192.3 (HGMD)	VUS	BS1, PS3, PP5	11 benign, 8 damaging
*TNNT2*	Troponin T2	Cardiac morphology	1q32	c.83C > T	p.Ala28Val	Missense	NM_001001430.2 NM_001276345.2 NM_000364.3 (ClinVar)	VUS	BS1, PP2, BP4	16 benign, 4 damaging
*TRPM4*	Transient receptor potential cation channel subfamily M member 4	Arrhythmogenic	19q13.33	c.1871 T > C	p.Val624Ala	Missense	NM_017636.3	VUS	PM2, BP4	18 benign, 3 damaging

*ACMG* American College of Medical Genetics, *VUS* variant of uncertain significance.

### Classification of variants

Variants were classified into five categories as defined by the American College of Medical Genetics and Genomics (ACMG) and the Association for Molecular Pathology^13^: *benign, likely benign, variants of uncertain significance (*VUS*)*; *likely pathogenic* and *pathogenic.*

### Statistical analyses

Distribution of data was evaluated using the Kolmogorov–Smirnov test. Not normally distributed variables are expressed as median and minimum/maximum. Categorical data are given as frequencies (n) and proportions (%). Continuous non-parametric data were compared using the Mann–Whitney U-test and Kruskal–Wallis test with Dunn's multiple comparison post-hoc test, respectively. Categorical non-parametric data were compared with Fisher’s Exact test. All reported *p*-values are two-sided, and level of significance was set at < 0.05. Statistical tests were performed with SPSS Statistics Version 26.0.0.0 (IBM Corporation, Armonk, NY, USA) and GraphPad Prism 8 for macOS Version 8.4.2 (GraphPad Software, LLC).

The study complied with the principles outlined in the Helsinki Declaration of 1975, as revised in 2013, and was approved by the institutional review board of the Ethics Committee at the Medical University of Vienna (*Reference Number 1852/2016*). All research was performed in accordance with relevant guidelines and regulations, and informed consent was obtained from all participants and/or their legal guardians in each case at the time of fetal sample acquisition for the post-mortem workup.

## Results

### Maternal and fetal baseline characteristics

From the total IUFD cohort, 16 cases of unexplained IUFD were included in this study (Fig. 1). Individual maternal and fetal characteristics of each case are shown in Supplementary Table 2. Median maternal age was 34 (16–44) years at time of delivery. Median maternal BMI was 21.7 (19.2–33.9) kg/m^2^. Two (12.5%) women were smokers and none consumed alcohol during pregnancy. Seven (43.8%) women were of Middle-European origin, four (25.0%) women were Turkish, two (12.5%) were from Eastern Europe, two (12.5%) were of African origin and one (6.3%) woman was originally from India. Consanguinity was present in two (12.5%) cases, one of Turkish, the other of Indian origin. Family history of cardiac problems was noted in one (6.2%) Caucasian woman, whose father had suffered myocardial infarction. Previous miscarriages were described in eight women. Previous stillbirths were noted in five women, one of whom had suffered two previous IUFDs.

The study cohort consisted of 12 (75.0%) male and four (25.0%) female fetuses. Median fetal gestational age at stillbirth was 34^+6^ (23^+2^–40^+5^) weeks. Median weight of stillborn fetuses was 2655 (486–3778) g with a median length of 49 (28–55) cm.

### Genetic variants

Of the 16 cases of unexplained IUFD, 14 (87.5%) fetuses were found to carry altogether 33 variants in 22 different cardio-specific genes (Supplementary Table 3). Five (15.2%) variants were classified as *likely benign* and 28 (84.8%) variants were considered as *variants of uncertain significance*, one of which was of likely benign (c.267C > G; *NEBL*) and another of likely pathogenic character (c.2224G > A *in MYH7*; Table 1).

Of the 22 genes involved, the majority of these (n = 14; 63.63%) were associated with cardiomyopathy (*DSP, JPH2, MYO6, MYPN, NDUFV2, NEBL, PRKAG2, RBM20, TNNT2, ILK, LAMA2, OBSCN, MYH7, MYOM1*); six (27.27%) were susceptibility genes for arrhythmogenic cardiac dysfunction (*AKAP9, KCNE1, SCN10A, TRPM4, ANK2, SCNN1A*) and two (9.09%) genes (*TBX5, DSG2*) were both arrhythmia and cardiomyopathy associated.

### Obstetric differences in fetuses with and without variants in cardio-specific genes

**Figure 2:**
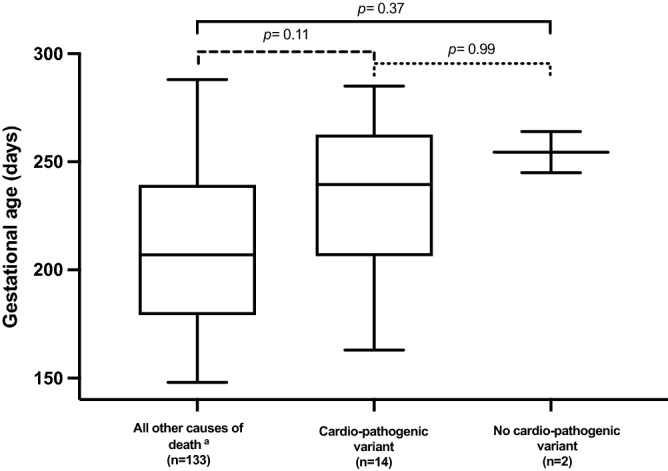
Comparison of median gestational age at time of stillbirth (in days) between fetuses with (n = 14) or without (n = 2) cardio-pathogenic variants and all cases of fetal death due to other causes (n = 133) as assessed by Kruskal–Wallis test with Dunn’s multiple comparisons. Superscript a: Causes of death (as per Tulip Classification): Placental dysfunction, cord complications, infection, “others”: e.g. fetal hydrops of unknown origin; maternal disease; fetal/maternal trauma. Excluded: congenital malformations (n = 32) and unknown because important information missing (n = 26).

No significant clinical differences in terms of maternal age, previous history of stillbirth, time of stillbirth or fetal sex were evident in fetuses with and without cardio-pathogenic variants. In comparison between our study subjects and the total IUFD cohort (*excluding* fetal congenital malformations and cases with important post-mortem information missing), we found no statistical difference regarding gestational age at stillbirth (Dunn's multiple comparison test; Fig. 2), yet, with regards to fetal weight (*p* = 0.001; Table 2).

**Table 2:** Maternal and fetal characteristics compared between explained and unexplained causes of fetal death.

Characteristics	Explained intrauterine fetal death^a^ (except fetal malformations and important information missing) (n = 133)	Unexplained intrauterine fetal death ^b^		*p*-value
		Variants detected in cardio-specific genes (n = 14)	No cardio-pathogenic variants detected (n = 2)	
Maternal age (years)	30 (17–44)	35 (25–44)	21 (16–25)	0.243^c^
Gravida (n)	2 (1–11)	4 (1–6)	1 (1–1)	**0.037**^c^
Para (n)	0 (0–9)	2 (0–4)	1 (1–1)	** < 0.001**^c^
BMI (kg/m^2^)	23.8 (15.6–45.3)	21.6 (19.2–33.9)	22.8 (21.8–23.8)	0.450^c^
Previous stillbirths (n)	0 (0–2)	0 (0–2)	1 (1–1)	**0.015**^c^
Previous miscarriages (n)	0 (0–3)	1 (0–3)	0 (0–0)	**0.003**^c^
Gestational age at stillbirth (days)	207 (148–288)	240 (163–285)	255 (245–264)	**0.013**^c^
Female (n; %)	69 (51.9)	4 (28.6)	0 (0.0)	0.068^d^
Male (n; %)	64 (48.1)	10 (71.4)	2 (100.0)	
Weight (g)	970 (180–4450)	2180 (486–3778)	2848 (2766–2930)	**0.001**^c^
Length (cm)	37 (20–55)	46 (28–55)	50 (49–51)	**0.002**^c^

*BMI* body mass index, *cm* centimeter, *g* gram.

^a^Causes of fetal death: placental dysfunction, cord complications, infection, “others”: e.g. fetal hydrops of unknown origin; maternal disease; fetal/maternal trauma. Excluded: congenital malformations (n = 32) and unknown because important information missing (n = 26).

^b^Study subjects of “unknown cause of fetal death despite thorough investigation” which underwent targeted sequencing.

^c^Mann–Whitney U test comparing non-parametric continuous data (median; minimum – maximum).

^d^Fisher’s exact test comparing non-parametric categorical data (n; %).

### Variants reassessment and reclassification

Between 2018 and 2020, all variants (n = 33) in the database were reassessed and reclassified. In 2018, variants of uncertain significance were denominated as VUS *with potential pathogenicity* (*VUSp*), when important information had been missing (e.g., family segregation) and variants were considered likely pathogenic.

None of the reclassified variants were upgraded over time. 33.3% (11/33) variants were downgraded, among which the majority of variants (6/11; 54.5%) were downgraded from *likely pathogenic* to VUS. 4 (36.4%) VUS and 1 (9.1%) *VUSp* were reclassified as *likely benign*.

66.6% (22/33) of the variants remained in the same category, all of which were classified as VUS, yet 54.5% (12/22) had been initially suspected as *VUSp.*

## Discussion

In this study involving ethnically diverse singleton fetuses after unexplained late IUFD, we aimed to investigate the prevalence of underlying cardio-pathogenic variants by targeted sequencing. Whilst we could not identify any putative cardio-pathogenic variant in two cases, the remaining 14 cases showed to harbor at least one variant reclassified as *likely benign* or *of uncertain significance* in genes involved in either heart rate regulation (causing potential e.g. LQTS, Brugada Syndrome, atrial fibrillation) and/or morphology (e.g. congenital dilated or hypertrophic cardiomyopathy). Although our sample size was too small to be conclusive, the scope over 122 cardiac genes revealed a high prevalence of *variants of unknown significance* in up to 75% fetuses, mainly involving genes which mutations most frequently would lead to cardiomyopathy.

A recent exome sequencing analysis in 246 unexplained stillborn fetuses revealed an underlying monogenetic cause in approximately 8.5% phenotypically normal fetuses.^14^ Also studies regarding cardio-pathogenic findings in otherwise unexplained stillbirths detected putative variants in 5.7% to 12.1% of cases^7,8^. In contrast to other authors, we found an incidence of 15.5% of *variants of uncertain significance* in genes contributing to LQTS, compared to the reported prevalence of over 50% of putative variants in previous investigations^15–17^.

Our study also illustrates the rapid change in variant classification over time: With the growing body of evidence within the recent years, in our cohort, six variants previously classified as *likely pathogenic* have been downgraded to *variants of uncertain signific*ance within 2 years. We described one VUS with likely pathogenic character (c.2224G > A *in MYH7*) due to positive family history^18–20^. According to the ACMG, “*a variant of uncertain significance should not be used in clinical decision making. Efforts to resolve the classification of the variant as pathogenic or benign should be undertaken. While this effort to reclassify the variant is underway, additional monitoring of the patient for the disorder in question may be prudent*”^13^. Although additional background data may strengthen or eliminate some of the *likely benign* or *likely pathogenic* variants in this study population, the high detection rate of *variants of uncertain significance* in the majority of cases of unexplained IUFD has not been described before. After all, this finding underlines the challenge following parallel sequencing of a high number of genes in interpreting and predicting the clinical impact of rare variants, especially when it comes to those potentially involved with genetic disorders that exhibit great variability in their penetrance and phenotypic manifestation. It is, therefore, important not to prematurely adjudicate ambiguous variants as potentially pathogenic, especially in cases with lack of further evidence and data such as parental comparative analysis prior to defining a diagnosis.

To our knowledge, this is the first comprehensive sequencing analysis of variants in 122 cardio-specific genes in a cohort strictly restricted to singleton intrauterine demised fetuses, hence excluding all cases of intrapartum and multiple fetal deaths or those with congenital malformations. Maternal, fetal and obstetrical data on all eligible stillbirth cases with conserved DNA between 2009 and 2017 had been collected to ensure solid adherence to inclusion and exclusion criteria to limit cases to unexplained IUFD only, as defined by the Tulip classification^11^.

However, our study is not devoid of study limitations. First and foremost, the relatively small cohort of 16 samples renders this analysis an underpowered pilot study, upon which future studies may build. Due to its retrospective characteristic of data collection, we were also unable to recruit affected family members for further segregation analysis. Furthermore, it is to be highlighted, that genetic analysis are probabilistic tests, rather than binary, and despite the identification of possible or probable mutations in cardiac genes, that have been associated with IUFD in literature before, the probability of pathogenesis in each IUFD case has to be regarded critically and interpreted under the light of clinical factors that are summed up in individuals’ “background genetic noise”, an environment in which underlying genetic changes may or may not migrate towards clinically apparent and relevant medical conditions. Also, we were unable to control for accuracy of the documentation of family history including potential cardiac diseases, as this was self-reported by the woman at the time of antenatal booking and therefore subject to recall bias. Our study cohort represents an ethnically inhomogeneous population group challenging the identification of possible ethnicity-specific putative variants due to its small study size.

Despite its study limitations as disclosed above, this pilot study supports the idea that genetic counseling and testing are important components of the post-mortem work-up in otherwise unexplained IUFD. Testing family members, foremost the affected parents should always be offered in order to evaluate their genetic predisposition and recurrence risk of IUFD based upon possible heterozygosity in arrhythmia- and cardiomyopathy-susceptible genes. The concomitant occurrence of multiple mutations in fetuses with IUFD, however, might challenge predictive counseling of parents in the context of pathogenic probability and recurrence risk in subsequent pregnancies, as every individual is a carrier of these variants to a certain degree that are subject to multifactorial silencing processes depending on one’s “genetic background noise”. After all, this field remains to be further explored, especially in cases of phenotypically normal fetuses of unexplained antepartum stillbirth.

Cardio-genetic pathologies might be a potentially underexplored etiology contributing to fetal death and therefore warrant further consideration within the frame of fetal post-mortem investigation with attentive appraisal for variants of uncertain significance.

## Supplementary Information

Supplementary Tables.
