# Supplementary material for: Cardio-pathogenic variants in unexplained intrauterine fetal death: a retrospective pilot study
Source: Sci Rep. 2021 Mar 24;11:6737. doi: 10.1038/s41598-021-85893-0 (PMC7991630; doi:10.1038/s41598-021-85893-0)
Supplement: Supplementary file 1 — Supplementary Tables. [file 41598_2021_85893_MOESM1_ESM.docx]

**Cardio-pathogenic variants in unexplained intrauterine fetal death –**

**A retrospective pilot study**

Dana A. Muin, MD, MRes^1*^, Martina Kollmann, MD, PhD^2^, Jasmin Blatterer, MSc^3^, Gregor Hoermann, MD, PhD^4,5^, Peter W. Husslein, MD^1^, Ingrid Lafer, MD^3,6^, Erwin Petek, MD, PhD^3^, Thomas Schwarzbraun, PhD^3,†^

^1^ Department of Obstetrics and Gynecology, Division of Fetomaternal Medicine, Medical University of Vienna, Austria

^2^ Department of Obstetrics and Gynecology, Division of Reproductive Medicine, Medical University of Graz, Austria

^3^ Institute of Human Genetics, Medical University of Graz, Austria

^4^ Department of Laboratory Medicine, Medical University of Vienna, Austria

^5^ Austria Central Institute of Medical and Chemical Laboratory Diagnostics, University Hospital Innsbruck, Innsbruck, Austria

^6^ Department of Internal Medicine, General Hospital Muerzzuschlag, Austria

^†^ Deceased.

***Corresponding Author:**

Dana Anaïs Muin, MD, MSc, MRes

Medical University of Vienna

Department of Obstetrics and Gynecology, Division of Fetomaternal Medicine

Waehringer Guertel 18–20, 1090 Vienna, Austria

*Tel:* +43 1 40400 28220, *Fax:* +43 1 40400 28620

*E-mail address:* [dana.muin@meduniwien.ac.at](mailto:dana.muin@meduniwien.ac.at)

**Supplementary Table 1.**

Cardiac phenotypes and their associated 122 cardiac genes analyzed in this study

| **CARDIAC PHENOTYPES** | **CARDIAC GENES** |
| --- | --- |
| Long-QT-Syndrome | *KCNH2, KCNQ1, SCN5A, AKAP9, ANK2, CACNA1C, CALM1, CALM3, CAV3, KCNE1, KCNE2, KCNE3, KCNJ2, KCNJ5, SCN4B, SNTA1* |
| Short-QT-Syndrome | *KCNH2, KCNJ2, KCNQ1, CACNA2D1, CACNB2* |
| Brugada-Syndrome | *SCN5A, SCN10A, ABCC9, CACNA1C, CACNA2D1, CACNB2, GPD1L, HCN4, KCND3, KCNE1L, KCNE3, KCNH2, KCNJ8, PKP2, RANGRF, SCN1B, SCN2B, SCN3B, SCNN1A, TRPM4* |
| Idiopathic ventricular and atrial fibrillation | *KCNA5, KCNE2, ABCC9, DSC2, GATA4, GATA6, GJA5, JPH2, KCND3, KCNE1, KCNE1L, KCNE3, KCNJ2, KCNJ8, KCNQ1, LMNA, NKX2-5, NKX2-6, NPPA, NUP155, SCN10A, SCN1B, SCN2B, SCN3B, SCN4B, SCN5A, TNNI3* |
| Atrioventricular blockage | *SCN5A, CALM1, DES, GJA5, HCN4, KCNA5, KCNE2, KCNJ2, KCNQ1, LMNA, NKX2-5, PRKAG2, SCN1B, TBX5, TRPM4* |
| Catecholaminergic polymorphic ventricular tachycardia | *CASQ2, KCNJ2, RYR2, CALM1, CALM3, LMNA, TRDN* |
| Arrhythmogenic right ventricular cardiomyopathy | *DES, DSC2, DSG2, DSP, JUP, PKP2, TMEM43, CTNNA3, LMNA, RYR2, TGFB3* |
| Hypertrophic cardiomyopathy | *MYBPC3, MYH7, TNNI3, TNNT2, TPM1, ACAD9, ACTC1, ACTN2, ANKRD1, CALR3, CAV3, COX15, CRYAB, CSRP3, CTF1, DES, FHL1, FHL2, FLNC, GAA, GLA, ILK, JPH2, KLF10, LAMA4, LAMP2, LDB3, MRPL3, MYH6, MYL2, MYL3, MYLK2, MYO6, MYOM1, MYOZ2, MYPN, NDUFV2, NEBL, NEXN, PDLIM3, PLN, PRKAG2, RAF1, RYR2, SCN5A, TCAP, TNNC1, TTN, TTR, VCL* |
| Dilatative cardiomyopathy | *LDB3, LMNA, MYBPC3, MYH7, PLN, TNNT2, ABCC9, ACTC1, ACTN2, ANKRD1, ATP2A2, BAG3, CHRM2, CRYAB, CSRP3, CTF1, DES, DMD, DNAJC19, DSC2, DSG2, DSP, DTNA, EMD, EYA4, FKTN, FLNC, ILK, LAMA2, LAMA4, LAMP2, MYH6, MYL2, MYL3, MYOM1, MYPN, NEBL, NEXN, OBSCN, PDLIM3, PSEN1, PSEN2, RAF1, RBM20, SCN5A, SDHA, SGCB, SGCD, SYNE1, SYNE2, TAZ, TBX20, TCAP, TMPO, TNNC1, TNNI3, TPM1, TTN, TTR, VCL* |
| Left ventricular non-compaction cardiomyopathy | *LDB3, ACAD9, ACTC1, CASQ2, DTNA, LMNA, MYBPC3, MYH7, NEBL, PRKAG2, RYR2, TAZ, TNNT2, TPM1* |
| Restrictive cardiomyopathy | *MYH7, TNNI3, ACTC1, CSRP3, DES, FLNC, GLA, LDB3, LMNA, MYBPC3, MYL2, MYOZ2, MYPN, PLN, TAZ, TCAP, TNNC1, TNNT2, TPM1, TTN* |
| Sudden Infant Death Syndrome, Sudden Unexplained Death Syndrome | *KCND3, KCNH2, KCNQ1, RYR2, SCN5A, AKAP9, ANK2* |

**Supplementary Table 2.**

Maternal and fetal characteristics per selected case (n=16)

| **ID** | **Ethnicity** | **Maternal Age (years)** | **Gravida (n)** | **Para (n)** | **Live births (n)** | **Stillbirths (n)** | **Miscarriages (n)** | **BMI (kg/m^2^)** | **Smoking** | **Alcohol** | **Consanguinity** | **GW** | **GD** | **Fetal sex** | **Weight (g)** | **Length (cm)** |
| --- | --- | --- | --- | --- | --- | --- | --- | --- | --- | --- | --- | --- | --- | --- | --- | --- |
| 1 | Turkish | 28 | 4 | 3 | 2 | 0 | 1 | 26,2 | no | no | yes | 40 | 5 | M | 3778 | 55 |
| 2 | Middle-European | 38 | 4 | 2 | 1 | 0 | 1 | 28,0 | no | no | no | 38 | 2 | M | 3260 | 51 |
| 3 | Indian | 27 | 1 | 1 | 0 | 0 | 0 | 30,3 | no | no | yes | 27 | 3 | F | 1060 | 37 |
| 4 | Middle-European | 35 | 1 | 1 | 0 | 0 | 0 | 35,3 | no | no | no | 36 | 1 | M | 3190 | 50 |
| 5 | Turkish | 34 | 5 | 2 | 1 | 0 | 3 | 27,9 | no | no | not disclosed | 39 | 6 | F | 3750 | 52 |
| 6 | Turkish | 27 | 4 | 3 | 2 | 0 | 1 | 19,5 | yes | no | not disclosed | 30 | 1 | M | 1130 | 37 |
| 7 | Middle-European | 44 | 5 | 2 | 2 | 0 | 2 | 22,6 | no | no | no | 23 | 2 | M | 486 | 28 |
| 8 | Eastern-European | 42 | 3 | 3 | 2 | 0 | 0 | 25,0 | no | no | no | 37 | 2 | M | 2710 | 49 |
| 9 | Middle-European | 25 | 3 | 2 | 1 | 0 | 1 | 21,9 | no | no | no | 36 | 6 | M | 2900 | 50 |
| 10 | African | 38 | 6 | 4 | 1 | 2 | 1 | 28,4 | no | no | no | 34 | 5 | M | 2600 | 50 |
| 11 | Eastern-European | 16 | 1 | 1 | 0 | 1 | 0 | 23,9 | no | no | no | 35 | 0 | M | 2766 | 49 |
| 12 | Middle-European | 35 | 1 | 0 | 0 | 0 | 0 | 23,4 | no | no | no | 33 | 0 | M | 1340 | 40 |
| 13 | Turkish | 29 | 2 | 1 | 1 | 1 | 0 | 26,0 | yes | no | no | 31 | 2 | M | 1546 | 42 |
| 14 | Middle-European | 44 | 5 | 4 | 4 | 1 | 0 | 26,3 | no | no | no | 26 | 4 | F | 740 | 35 |
| 15 | Middle-European | 25 | 1 | 1 | 0 | 1 | 0 | 21,8 | no | no | no | 37 | 5 | M | 2930 | 51 |
| 16 | African | 34 | 5 | 3 | 3 | 0 | 1 | 25,7 | no | no | no | 33 | 5 | F | 1760 | 41 |

*Abbreviations:* BMI, Body Mass Index; cm, centimeter; F, female; g, gram; GD, gestational day; GW, gestational week; ID, Identity number; M, male.

**Supplementary Table 3.**

Summary of putative cardio-pathogenic variants in 16 fetuses after otherwise unexplained intrauterine fetal death

Tabelle 1

| **ID** | **GW** | **GD** | **Sex** | **Location** | **Nucleotide Change** | **Amino Acid Change** | **Effect** | **Annotation** | **Gene** | **Gene description** | **Primary dysfunction** | **Cardiac phenotype** | **ACMG Designation** | **ACMG Criteria** |
| --- | --- | --- | --- | --- | --- | --- | --- | --- | --- | --- | --- | --- | --- | --- |
| **1** | 40 | 5 | Male | 21q22.12 | c.200G>A | p.Arg67His | missense | NM_001270402.1 NM_000219.6 (HGMD; ClinVar) | ***KCNE1*** | Potassium voltage gated channel | Arrhythmogenic | LQTS | VUS | PM2, PM5, PP3 |
| **2** | 38 | 2 | Male | 1q32 | c.83C>T | p.Ala28Val | missense | NM_001001430.2 NM_001276345.2 bzw. NM_000364.3 (ClinVar) | ***TNNT2*** | Troponin T2, cardiac type | Cardiac morphology | HCM | VUS | BS1, PP2, BP4 |
|  |  |  |  |  |  |  |  |  |  |  |  | DCM |  |  |
|  |  |  |  |  |  |  |  |  |  |  |  | RCM |  |  |
|  |  |  |  |  |  |  |  |  |  |  |  | LVNC |  |  |
|  |  |  |  | 7q21-q22 | c.763A>C | p.Thr255Pro | missense | NM_005751.4 | ***AKAP9*** | kinase anchor protein | Arrhythmogenic | LQTS | VUS | PM2 |
|  |  |  |  | 18q12.1 | c.3205A>G | p.Met1069Val | missense | NM_001943.3 | ***DSG2*** | Desmoglein 2 | Cardiac morphology, arryhthmogenic | ARVC | VUS | PM2, BP4 |
|  |  |  |  |  |  |  |  |  |  |  |  | DCM |  |  |
| **3** | 27 | 3 | Female | 12p13 | c.752G>C | p.Arg251Thr | missense | NM_001159576.1 NM_001038.5 (HGMD, ClinVar) | ***SCNN1A*** | Sodium channel, non-voltage gated 1 | Arrhythmogenic | (Brugada) | VUS | BP4, PM2 |
| **4** | 36 | 1 | Male | 19q13.33 | c.1871T>C | p.Val624Ala | missense | NM_017636.3 | ***TRPM4*** | Transient receptor potential cation channel | Arrhythmogenic | Brugada | VUS | PM2, BP4 |
| **5** | 39 | 6 | Female | 14q12 | c.3200T>C | p.Met1067Thr | missense | NM_000257.2 | ***MYH7*** | Myosin, heavy polypeptide 7 | Cardiac morphology | HCM | VUS | PM2, PP2, PP3 |
|  |  |  |  |  |  |  |  |  |  |  |  | DCM |  |  |
|  |  |  |  |  |  |  |  |  |  |  |  | LVNC |  |  |
|  |  |  |  | 18p11.31-p11.2 | c.604G>A | p.Glu202Lys | missense | NM_021074.4 | ***NDUFV2*** | NADH dehydrogenase (ubiquinone) flavoprotein 2 | Cardiac morphology | HCM | VUS | BP4 |
| **6** | 30 | 1 | Male | 12p13 | c.1618G>A | p.Val540Met | missense | NM_001159576.1 | ***SCNN1A*** | Sodium channel, non-voltage gated 1 | Arrhythmogenic | (Brugada) | VUS | BP4, PS3 |
|  |  |  |  | 6q22-q23 | c.6598C>T | p.Arg2200Cys | missense | NM_000426.3 | ***LAMA2*** | Laminin alpha 2 (merosin) | Cardiac morphology | (DCM) | VUS | PP3, BP1 |
|  |  |  |  | 10q21.3 | c.3589G>A | p.Gly1197Ser | missense | NM_001256267.1 | ***MYPN*** | Myopalladin | Cardiac morphology | DCM | VUS | PM2, PP3 |
|  |  |  |  |  |  |  |  |  |  |  |  | HCM |  |  |
|  |  |  |  |  |  |  |  |  |  |  |  | RCM |  |  |
| **7** | 23 | 2 | Male | 18p11.31 | c.4814C>T | p.Ser1605Leu | missense | NM_003803.3 | ***MYOM1*** | Myomesin 1 | Cardiac morphology | HCM | VUS |  |
| **8** | 37 | 2 | Male | 10p12 | c.267C>G | p.Tyr89Ter | stop | NM_006393.2 | ***NEBL*** | Nebulette | Cardiac morphology | DCM | VUS/Likely benign | BS1 |
|  |  |  |  | 12q24.1 | c.1115C>T | p.Ser372Leu | missense | NM_181486.2 NM_000192.3 (HGMD) | ***TBX5*** | T-box 5 | Cardiac morphology, arryhthmogenic | Holt-Oram syndrome | VUS | BS1, PS3, PP5 |
|  |  |  |  |  |  |  |  |  |  |  |  | AF |  |  |
|  |  |  |  | 6q13 | c.2307C>G | p.Ile769Met | missense | NM_004999.3 | ***MYO6*** | Myosin VI | Cardiac morphology | HCM | VUS | PM2, BP4 |
| **9** | 36 | 6 | Male | 14q12 | c.2224G>A | p.Ala742Thr | missense | NM_000257.2 | ***MYH7*** | Myosin, heavy polypeptide 7 | Cardiac morphology | HCM | VUS/Likely pathogenic | PM1, PM2, PP2, BP4 |
|  |  |  |  |  |  |  |  |  |  |  |  | DCM |  |  |
|  |  |  |  |  |  |  |  |  |  |  |  | LVNC |  |  |
|  |  |  |  | 4q25-q27 | c.7436A>G | p.Lys2479Arg | missense | NM_001148.4 | ***ANK2*** | Ankyrin 2, neuronal | Arrhythmogenic | LQTS | VUS |  |
|  |  |  |  |  |  |  |  |  |  |  |  | SUDS |  |  |
|  |  |  |  | 1q42.13 | c.23631A>T | p.Arg7877Ser | missense | NM_001098623.1 (obscntv2 in HGMD) NM_001271223.2 (ClinVar) NM_052843.3 (OBSCN in HGMD) | ***OBSCN*** | Obscurin, cytoskeletal calmodulin and titin-interacting RhoGEF | Cardiac morphology | DCM | VUS | PM2, BP4 |
| **10** | 34 | 5 | Male | 6p24 | c.5513G>A | p.Arg1838His | missense | NM_004415.2 | ***DSP*** | Desmoplakin | Cardiac morphology | ARVC | VUS | BP1 |
|  |  |  |  |  |  |  |  |  |  |  |  | DCM |  |  |
|  |  |  |  | 18p11.31 | c.5045dupA | p.Lys1683GlufsTer16 | Duplication (frameshift | NM_003803.3 | ***MYOM1*** | Myomesin 1 | Cardiac morphology | HCM | Likely benign | BS1, BP6 |
|  |  |  |  | 10q25.2 | c.1451C>T | p.Thr484Ile | missense | NM_001134363.1 | ***RBM20*** | RNA binding motif protein 20 | Cardiac morphology | DCM | Likely benign | BS1, BP4, PP2 |
|  |  |  |  | 4q25-q27 | c.11459G>A | p.Arg3820Gln | missense | NM_001148.4 | ***ANK2*** | Ankyrin 2, neuronal | Arrhythmogenic | LQTS | Likely benign | BS1, BP6, BP4 |
|  |  |  |  |  |  |  |  |  |  |  |  | SUDS |  |  |
| **11** | 35 | 0 | Male | *-* | *-* | *-* | *-* | - | *-* | *-* | *-* | *-* | *-* | *-* |
| **12** | 33 | 0 | Male | 11p15.5-p15.4 | c.521G>A | p.Arg174His | missense | NM_004517.2 | ***ILK*** | Integrin-linked kinase | Cardiac morphology | DCM | VUS |  |
|  |  |  |  |  |  |  |  |  |  |  |  | HCM |  |  |
| **13** | 31 | 2 | Male | 20q13.12 | c.572C>G | p.Pro191Arg | missense | NM_020433.4 | ***JPH2*** | Junctophilin 2 | Cardiac morphology | HCM | Likely benign | BS1, BP4 |
|  |  |  |  |  |  |  |  |  |  |  |  | SUDS |  |  |
|  |  |  |  | 4q25-q27 | c.9520A>G | p.Thr3174Ala | missense | NM_001148.4 | ***ANK2*** | Ankyrin 2, neuronal | Arrhythmogenic | LQTS | VUS | PM2, BP4 |
|  |  |  |  |  |  |  |  |  |  |  |  | SUDS |  |  |
| **14** | 26 | 4 | Female | 3p22.2 | c.3674T>C | p.Ile1225Thr | missense | NM_006514.2 | ***SCN10A*** | Sodium channel, voltage-gated, | Arrhythmogenic | Brugada | Likely benign | PP3, BS1, BS2 |
|  |  |  |  |  |  |  |  |  |  |  |  |  |  |  |
|  |  |  |  | 1q42.13 | c.4393G>A | p.Glu1465Lys | missense | NM_001098623.1 | ***OBSCN*** | Obscurin, cytoskeletal calmodulin and titin-interacting RhoGEF | Cardiac morphology | DCM | VUS | BS1, BP4 |
| **15** | 37 | 5 | Male | *-* | *-* | *-* | *-* | - | *-* | *-* | *-* | *-* | *-* | *-* |
| **16** | 33 | 5 | Female | 1q42.13 | c.7457T>C | p.Val2486Ala | missense | NM_001098623.1 | ***OBSCN*** | Obscurin, cytoskeletal calmodulin and titin-interacting RhoGEF | Cardiac morphology | DCM | VUS |  |
|  |  |  |  | 1q42.13 | c.9545C>T | p.Ala3182Val | missense | NM_001098623.1 | ***OBSCN*** | Obscurin, cytoskeletal calmodulin and titin-interacting RhoGEF | Cardiac morphology | DCM | VUS | BP4 |
|  |  |  |  | 6q22-q23 | c.3778G>A | p.Glu1260Lys | missense | NM_000426.3 | ***LAMA2*** | aminin alpha 2 (merosin) | Cardiac morphology | (DCM) | VUS | PM2, BP1 |
|  |  |  |  | 7q36.1 | c.1475T>A | p.Ile492Asn | missense | NM_016203.3 | ***PRKAG2*** | Protein kinase, AMP-activated | Cardiac morphology | DM | VUS | PP2, PP3 |
|  |  |  |  |  |  |  |  |  |  |  |  | HCM |  |  |
|  |  |  |  | 11p15.5-p15.4 | c.684T>G | p.Ser228Arg | missense | NM_004517.2 | ***ILK*** | Integrin-linked kinase | Cardiac morphology | DCM | VUS | PM2 |
|  |  |  |  |  |  |  |  |  |  |  |  | HCM |  |  |
|  |  |  |  | 18q12.1 | c.880A>G | p.Lys294Glu | missense | NM_001943.3 | ***DSG2*** | Desmoglein 2 | Cardiac morphology, arryhthmogenic | ARVC | VUS |  |
|  |  |  |  |  |  |  |  |  |  |  |  | DCM |  |  |

*Abbreviations*: ACMG, American College of Medical Genetics; AF, atrial fibrillation; ARVC, arrhythmogenic right ventricular cardiomyopathy; DCM, dilated cardiomyopathy; GD, gestational day; GW, gestational week; HCM, hypertrophic cardiomyopathy; ID, Identity number; LQTS, Long-QT-Syndrome; SUDS, sudden unexplained death syndrome; VUS, variant of uncertain significance
